# An interview with Professor Konasale Prasad and Professor Jeffrey Bishop: progress in psychoradiology revolutionizes the diagnostic and therapeutic landscape of mental disorders

**DOI:** 10.1093/psyrad/kkaf016

**Published:** 2025-06-03

**Authors:** Long-Biao Cui, Lan Wang, Shuang Luo

**Affiliations:** Research Fellow at Shaanxi Provincial Key Laboratory of Clinical Genetics, Fourth Military Medical University, Xi'an 710032, China; Research Fellow at Schizophrenia Imaging Lab, Xijing 986 Hospital, Fourth Military Medical University, Xi'an 710054, China; Science and news editors at the editorial office of *Psychoradiology* in Chengdu, China; Science and news editors at the editorial office of *Psychoradiology* in Chengdu, China

## Abstract

Professor Jeffrey Bishop from the University of Minnesota and Professor Konasale Prasad from the University of Pittsburgh were invited to attend the ISMRM-Endorsed Workshop on MR for Psychiatry in Chengdu, China, from 20 to 22 July 2024. Professor Bishop and Professor Prasad delivered lectures on the molecular and neuro-mechanism of schizophrenia respectively during the session titled “Exploring Schizophrenia with MRI” on the morning of 21 July. Their presentations were met with great enthusiasm and sparked lively discussions among the participants. Following the conference, the *Psychoradiology* journal interviewed Professors Prasad and Bishop. In the interview, they narrated their personal journeys into the research field and unanimously agreed that psychoradiological techniques have brought a revolutionary change in the characterization of phenotypes with potential future implications for facilitating diagnosis, prognosis, and treatment strategies of mental disorders. They also noted that the field is now facing technological challenges and resource constraints, and that defining mental illnesses biologically and achieving precision treatment will be significant opportunities and challenges in the future. They highlighted the importance of interdisciplinary collaboration, believing it fosters in-depth dialogue across various domains. Additionally, they encouraged young researchers to maintain perseverance and patience in the long run of scientific research, aligning their goals effectively with practice.

## Progress in psychoradiology revolutionizes the diagnostic and therapeutic landscape of mental sisorders

1. ***Psychoradiology:* To inspire young researchers, could you please share with us how you entered your research field?**

**Prasad:** I initially became interested in schizophrenia for its distinctive cognitive traits, such as delusions and disorganized thinking. My early research was basically a survey and a review paper focusing on the first-rank symptoms of schizophrenia, as described by Kurt Schneider in the 1930s. After relocating to the USA, I shifted my focus to delusions, utilizing neuroimaging to explore the role of the entorhinal cortex and parahippocampal gyrus in their development. This experience drew me into neuroimaging because it allowed me to study the brain without waiting for post-mortem tissue analysis. The non-invasive, comprehensive, and sustainable data provided by neuroimaging captivated me, prompting my venture into this domain.

**Bishop:** My early fascination with genetics originated from undergraduate research and formal training. Working in a lab with a molecular neuroscientist who was also a neurologist sparked my interest in clinical neuroscience. Consequently, I pursued clinically focused training while continuing laboratory research and gained experience in adult psychiatric facilities. During my postgraduate training, I was a clinical team member of a longitudinal Assertive Community Treatment program, making house visits to patients with severe mental illness. I managed pharmacotherapy and medication management, observing the variability in patients’ organization and response to medications. This experience prompted me to apply genetics to better understand individual differences, leading me into pharmacogenetics. After training, I joined the University of Illinois Chicago, where collaboration with Dr John Sweeney helped establish my research in the integration of genetics and therapeutic outcomes.

2. ***Psychoradiology :* What initially ignited your passion for your particular research area, and how has that enthusiasm evolved and influenced your academic pursuits over time?**

**Prasad:** My passion for research ignited during my residency, leading me to question traditional clinical practices and seek innovative approaches, such as genetic markers for treatment. Despite the financial trade-offs of reduced clinical work, I remain committed to translational research, collaborating with a private company to apply my findings in real-world settings. Beyond academic practice, my ultimate aim is to make these advancements accessible to patients, which is the core reason for my dedication to research.

**Bishop:** What truly propelled my research passion forward was my fellowship, during which I worked on a project involving antidepressant side effects. Through surveys and genetic studies, I have overseen patient interactions, uncovering that even “safe” medications can have significant side effects. Clinical work in my small hometown further enhanced my interest in pursuing research that has tangible, positive impacts on the community.

3. ***Psychoradiology :* Given the substantial achievements you have garnered in your research field, what would you consider to be the most significant discovery or contribution of your research?**

**Prasad:** Our research on the impact of herpes simplex virus 1 (HSV-1) on schizophrenia revealed a correlation between the virus and cognitive deficits, rather than the disease itself. Imaging studies showed that HSV-1-exposed patients had smaller prefrontal cortexes, correlating with cognitive impairments. We conducted a small-scale clinical trial showing cognitive improvement with valacyclovir treatment, though inconsistent across domains, particularly in early-onset cases. Despite these initial promising results, securing funding for broader studies was difficult. The research gained interest from an Alzheimer's group, leading to a related clinical trial. They conducted a clinical trial, which is now near completion, and published the protocol in *BMJ Open*. Extrapolating animal model results to humans requires more caution.

**Bishop:** I have been involved in both discovery and clinically applied psychopharmacology research, investigating genomic underpinnings of illness pathophysiology and its treatment response. Initially focused on genetic discovery, part of my work has now evolved to refining clinically available pharmacogenomic tests and establishing treatment guidelines for incorporating these results into clinical care. We have recently completed one guideline for antidepressant medications and are currently working on another for antipsychotics. Converting discovery work into something clinically useful is particularly rewarding, and it is one accomplishment I am proud of.

4. ***Psychoradiology*: With the prospects for scientific research changing dramatically, what is your perspective on the developments in this field over the past few decades?**

**Prasad:** Neuroimaging technology is rapidly evolving, with new developments emerging every year. With the advent of 7T magentic resonance imaging (MRI) and new analysis methods, the potential for MRI-guided diagnostics and prognostics has greatly increased. One challenge with MRI is that many patients are uncomfortable with the enclosed space of traditional MRI machines. To address this, open MRIs and head-only MRIs are being developed specifically for neuroscience applications, making the process more acceptable to patients. Additionally, the introduction of portable, low-tesla machines, like the 0.5T device, facilitates imaging in non-clinical settings. Comparative studies show that this equipment offers imaging quality comparable to higher-tesla machines. Furthermore, the emergence of pharmacological imaging techniques, including xenon and carbon-13, marks significant progress, though resolution issues remain.

**Bishop:** The integration of diverse data sources marks a significant leap in research, shifting from isolated studies of single genetic variations to a more holistic approach. With increased sample sizes, enhanced deep phenotyping, including neuroimaging, and a deeper understanding of genomics, we are more able to tackle complex questions. Factors such as medication adherence, behavior, and early life experiences broaden our scope, offering a more complete and powerful perspective than ever before.

5. ***Psychoradiology*: As new imaging technologies continue to emerge and mature, which imaging technology do you see as the most notable advancement that will have a huge impact on your field?**

**Prasad:** Imaging technology must be selected based on the specific clinical question, with new modalities continually emerging to meet diverse needs. For instance, in geriatric medicine and psychiatry, characterizing microvascular changes in the elderly often requires specialized methods such as susceptibility-weighted imaging (SWI). Back in the 1990s, SWI signals used to be thrown away as noise during signal processing. However, Mark Harkie and others identified the value in previously discarded data and then developed this method, which is now widely used to detect iron deposits and microvascular changes with remarkable precision. Another example is the study of the glymphatic system. Scanning every modality for a patient can be challenging, as some individuals cannot tolerate extended scanning times. To address this, we need to develop rapid pulse sequences and select the most relevant imaging modalities for each patient. This ensures efficient and effective use of imaging technologies without overwhelming the patient.

**Bishop:** I agree with Prasad's perspective. There are opportunities for all of them, especially with the development of new therapeutics for schizophrenia. One potential area is neurotransmitter receptor profiling using positron emission tomography (PET) imaging, although this method has not yet been widely applied to other neurotherapeutics. Additionally, fingerprinting technology, which involves sparse sampling and condensed data representation, is promising for capturing signals and filling in gaps through database informatics. This offers a more efficient and rapid method for data collection.

6. ***Psychoradiology*: In the next 5 years, what do you think will be the new challenges and opportunities in the field?**

**Prasad:** There are many challenges and opportunities in defining psychiatric disorders biologically. Without biological parameters, we will continue to struggle with heterogeneity. If we can biologically define schizophrenia, we can identify different subtypes (type 1, type 2, etc.) and tailor medications accordingly. The same applies to bipolar disorder, major depression, obsessive–ompulsive disorder (OCD), and post-traumatic stress disorder (PTSD). In the case of PTSD, only a small proportion of individuals exposed to stress or trauma develop the disorder. Understanding the pathophysiology of PTSD and what protects the large percentage who do not develop PTSD could lead to preventative strategies and new treatments.

**Bishop:** Precision therapeutics encompasses both opportunities and challenges. From a drug development perspective, pharmaceutical companies lack incentives to create drugs for small subgroups, such as the few per cent of individuals with specific subtypes of schizophrenia. The industry focuses on broader populations, leaving it to academic researchers to address these niche areas. As Prasad mentioned, funding streams often do not support this more detailed work. Researchers often juggle multiple roles for their labs, from the business-world equivalents of chief executive officer and chief financial officer to lab maintenance, adding to the challenge. Therefore, resource limitations, coupled with scientific hurdles, are parallel challenges that need to be addressed.

## Interdisciplinary and international collaboration fosters dialogue across different fields

7. ***Psychoradiology*: What is the role of interdisciplinary collaboration in advancing scientific progress? Can you share with us one of your successful experiences of interdisciplinary collaboration?**

**Prasad:** In my lab, a diverse team of mathematicians, physicists, statisticians, neuroscientists, clinicians, and engineers collaborates on a regular basis. My role is to act as an integrator, translating complex mathematical, statistical, and engineering concepts into clinically relevant terms. This fosters dialogue and addresses challenges, which in turn catalyzes the emergence of innovative ideas.

**Bishop:** My research has always been highly interdisciplinary, integrating psychology, neuropsychology, psychiatry, and pharmacy. For instance, our lab involves basic pharmacologists, psychiatrists, and pharmacists, reflecting a collaborative, interprofessional approach. Throughout my career, I have consistently worked in a collaborative environment. At many points, I have spent more time with psychiatrists and neuropsychologists than with professionals in my own field.

8. ***Psychoradiology*: How do you perceive the significance of international cooperation in the context of scientific research, especially within the realm of globalization and internationalization?**

**Prasad:** I engaged in international collaboration, mentoring early-career Indian researchers on multi-center grants and partnering with a mathematician in France. I also have started to engage with Dr Su Lui's group as well. Valuable resources, such as the UK Biobank, provide data to qualified researchers at a low cost and are sometimes for free if researchers are from low-income countries. Similarly, the USA has the ABCD and other repositories, which are open for use by anyone. We hope to see more of this kind of international collaboration from Asian countries as well. Without such efforts, studies often reflect Western populations and may not generalize well to other regions. However, it depends on government decisions and investment in these initiatives in the respective countries.

**Bishop:** Governmental elements and communication styles can affect international collaboration. I have been working with Professor Lui Su's group for several years, and the guideline groups I am involved with are now intentionally international. Each of these collaborations is rewarding.

9. ***Psychoradiology*: Conveying research to the public is also an indispensable part of scientists’ work. How do you convey your research findings to the public? Besides conferences and academic publications, do you have other methods to share your findings with the public?**

**Prasad:** That is an art. It is crucial to convey information clearly. What we discuss in the lab may seem unintelligible to most people, but when presenting at conferences, the level of complexity is different. For presentations to patients and families, I simplify the information to ensure it is understandable to the public with different educational backgrounds. Similarly, when recruiting patients and obtaining informed consent, I focus on explaining the research in a clear and accessible way. It is about balancing detail with simplicity and emphasizing the risks and benefits to participants and the public over the intricate science. Conveying information to clinicians can be challenging, particularly because they have limited time and a high patient volume. Given these constraints, it is important to provide information efficiently. For this reason, we organize large-scale public education events, such as the annual schizophrenia and bipolar disorder conferences, where patients, clinicians, and psychiatrists gather. At these conferences, we present information at a level appropriate for the audience. For instance, at meetings like those of the American Psychiatric Association, top scientists provide an overview of the field to educate clinicians. These presentations are designed to be accessible, without expecting clinicians to have advanced methodological expertise.

**Bishop:** I occasionally present to patient advocacy groups, such as the National Alliance on Mental Illness, where I discuss elements of precision medicine with patients and their families. While I am not very active on social media, which has not been a good fit for me, I understand its value for dissemination within clinical groups.

## Suggestions for young scientists in the field and insights on career development

10. ***Psychoradiology*: What are your suggestions for young researchers when they consider their research directions and professional path?**

**Prasad:** I value the enthusiasm of undergraduate students who are eager for research. However, many are unaware that research is a marathon, not a sprint, demanding lifelong dedication and resilience. As scientists, we must embrace the inherent challenges, including rejections and critiques, which are impersonal and essential for growth. Critiques raise important questions rather than targeting individuals in most circumstances. Having experienced the review process as both an applicant and a panelist, I would like to tell young researchers that the research process itself is human-driven, self-motivated, and imperfect.

**Bishop:** Understanding the broader purpose of one's challenging journey serves as a key motivator to overcome obstacles. I have also come to appreciate the broad spectrum of scholarly activities beyond traditional research. This spectrum includes full-time research program investigators, those who combine clinical practice with clinical trials, and those involved in disseminating case reports or clinical education. Research is not a singular path. There is a place for various interests within this field. By broadening their perspective and finding the right match between their motivations and their role, individuals can better align their goals with their meaningful contributions.

11. ***Psychoradiology*: Now we would like to talk about some more relaxed and interesting topics. Could you share with us some of your personal stories about the challenges you encountered when you entered your research field, and how you overcame them?**

**Prasad:** I used to describe myself as being like a leech—sticking to my objectives and continuously working towards them. I realized that staying in India for too long could lead to burnout, so I left India in search of opportunities to realize my goals, despite the uncertainty. I initially landed in England, but I soon shifted my focus to the USA, ultimately settling in Pittsburgh. I found support through my mentors. My mentors noticed my struggles as well as my passion for research and connected me with opportunities in Pittsburgh and outside of the University. Personally, the key is perseverance, seeking support, and connecting with the right individuals. If one person does not help, you should move on to the next person. You should bear in mind that rejecting what is not right for you is crucial to finding where you truly belong.

**Bishop:** My first paper as the first author was a schizophrenia-oriented study which examined the equality of treatments for women receiving osteoporosis care, a clinically relevant topic. The reviews I received were longer than the paper itself, which was quite overwhelming. The journal, *Pharmacotherapy*, is multidisciplinary. Although the reviews included extensive grammatical edits, the editor recognized the importance of the paper. He highlighted the essential feedback, perhaps realizing that I was a young trainee. My supervisor also helped me navigate the reviews, turning the experience into a valuable learning opportunity. It is better to improve your writing over time, especially as you review more and understand how your work might be interpreted. This helps you enhance the clarity of your message and ensure that the key points of your dissemination are prominent. I have learned to refine my approach and improve the structure of my writing. Finding your own style and continuously honing it is part of the process.

12. ***Psychoradiology*: Have you written any scientific books that make your research accessible to the general public?**

**Prasad:** When I retire, I might consider writing a book. However, writing is very time-consuming, and there are already many excellent books available in the USA on topics such as schizophrenia and bipolar disorder, written by prominent scientists. Given the abundance of existing resources, it is challenging to see where another book would fit in.

**Bishop:** I have contributed to several book chapters for a medical textbook. The textbook is used for pharmacy training, which covers topics such as patient assessments and the integration of a holistic approach.

13. ***Psychoradiology*: The open access process significantly impacts the dissemination of knowledge and the accessibility of research findings. What is your opinion on its role in academia?**

**Prasad:** Facing financial constraints with open access publishing, I have navigated the academic landscape by leveraging institutional contracts that waive fees, as seen with my submission to *Human Brain Mapping*. Due to grant limitations and increasing operational costs, I avoid open access fees by sharing publicly available papers directly via email and utilizing repositories such as the US National Library of Medicine, which mandates free access post-embargo. I also resort to uploading my work on platforms such as bioRxiv and my lab website, ensuring accessibility without the associated costs. This strategy maintains my outreach and citation impact without the financial burden of traditional open access models.

**Bishop:** I am not entirely convinced that open access is wholly beneficial or detrimental. It appears to encompass both advantages and drawbacks. In my experience, open access publications tend to receive more citations and greater visibility due to their wider availability. However, the financial aspect introduces a challenge in that researchers who are able to cover publication costs enjoy greater ease in disseminating their work, while those with limited funding might find publication more challenging. Additionally, the transparency of open access is a clear advantage: paying the fee is a straightforward transaction. Conversely, traditional publishing can involve unexpected costs, such as those for color images, which are often disclosed only after acceptance, obligating authors to pay. Thus, the upfront nature of open access fees promotes transparency and predictability in the publication process.

14. ***Psychoradiology*: This is the fourth year for our journal. Do you have any suggestions for increasing our influence in the future?**

**Prasad:** This is very challenging because the environment for journal development is highly competitive. There are too many periodicals and journals available. At least from my perspective, another problem is that I generally do not publish in open access journals, mainly because I have limited funds to cover publication fees.

**Bishop:** I have been involved in *Schizophrenia Research* since 2017 and have served on the editorial board of *Pharmacotherapy* for a long time. I have heard various conversations about the challenges of finding peer reviewers and the importance of impact factors. I suggest that your journal could invite prominent scholars, even within your local community, to contribute some conceptual and integrative pieces. This can help boost citation impact. Additionally, an international editorial board can encourage contributions from global experts.

### Expert's information

**Figure 1:**
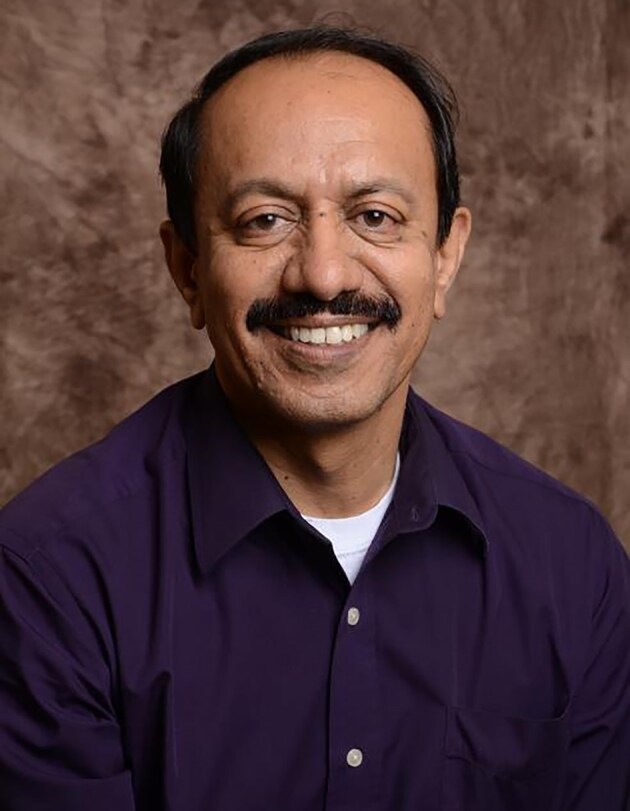


Dr Konasale Prasad is an Associate Professor of Psychiatry and Bioengineering at the University of Pittsburgh and serves as an Associate Editor for *Schizophrenia Research*. He is the Director and Principal Investigator at the CONCEPT (Connecting Outcomes Networks & Cognition for Early Psychosis Therapeutics) Lab (https://www.conceptlab.pitt.edu). Board-certified by the American Board of Psychiatry and Neurology, Dr Prasad is also a Fellow of the American College of Neuropsychopharmacology (ACNP) and a member of the Society of Biological Psychiatry (SOBP). Dr Prasad's research focuses on the neurobiology and genetics of schizophrenia and at-risk mental states for psychotic disorders. He has published approximately 140 papers in peer-reviewed journals and authored over 18 book chapters. Dr Prasad has received notable awards, including the Fellowship Award from the Society of Biological Psychiatry and the Outstanding Resident Award from the National Institute of Mental Health.

**Figure 2:**
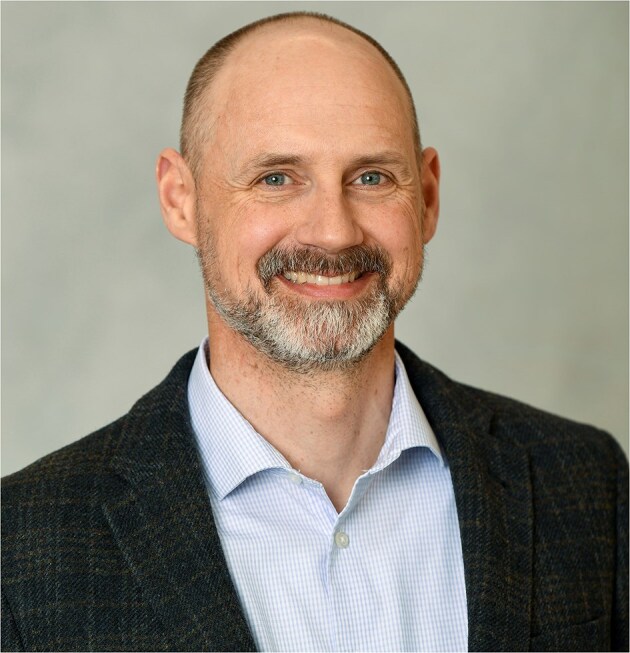


Dr Jeffrey Bishop is a Professor at the University of Minnesota. He served as an Associate Editor for *Schizophrenia Research* from 2017 to 2024. He is a Fellow of the American College of Clinical Pharmacy and has received several prestigious awards, including the National Institute of Mental Health (NIMH) Research Career Development Award, the College of Psychiatric and Neurologic Pharmacists New Investigator Award, and the American College of Clinical Pharmacy Career Development Award. Dr Bishop specializes in psychopharmacology and pharmacogenomics, focusing on the genetic relationships influencing symptom improvement, side effects, and cognitive effects of medications. He has published over 160 peer-reviewed papers in journals such as *Molecular Psychiatry, Brain Behavior Immunity*, and *Neuropsychopharmacology*, with more than 9000 citations on Google Scholar.

## List of Abbreviations

ABCD: Adolescent Brain Cognitive Development
BMJ: British Medical Journal
HSV-1: Herpes Simplex Virus Type 1
MRI: Magnetic Resonance Imaging
OCD: Obsessive-Compulsive Disorder
PET: Positron Emission Tomography
PTSD: Post-Traumatic Stress Disorder
SWI: Susceptibility-Weighted Imaging
US: United States
UK: United Kingdom

